# Associations of circulating folate, vitamin B12 and homocysteine concentrations in early pregnancy and cord blood with epigenetic gestational age: the Generation R Study

**DOI:** 10.1186/s13148-021-01065-x

**Published:** 2021-04-29

**Authors:** Giulietta S. Monasso, Leanne K. Küpers, Vincent W. V. Jaddoe, Sandra G. Heil, Janine F. Felix

**Affiliations:** 1 The Generation R Study Group (Na-2918), Erasmus MC, University Medical Center Rotterdam, Rotterdam, The Netherlands; 2 Department of Pediatrics, Erasmus MC, University Medical Center Rotterdam, Rotterdam, The Netherlands; 3 Division of Human Nutrition and Health, Wageningen University, Wageningen, The Netherlands; 4 Department of Clinical Chemistry, Erasmus MC, University Medical Center Rotterdam, Rotterdam, the Netherlands

**Keywords:** Folate, Holotranscobalamin, Vitamin B12, Homocysteine, DNA methylation, Epigenetic clock, Gestational age, Cohort study, Children

## Abstract

**Background:**

Circulating folate, vitamin B12 and homocysteine concentrations during fetal development have been associated with health outcomes in childhood. Changes in fetal DNA methylation may be an underlying mechanism. This may be reflected in altered epigenetic aging of the fetus, as compared to chronological aging. The difference between gestational age derived in clinical practice and gestational age predicted from neonatal DNA methylation data is referred to as gestational age acceleration. Differences in circulating folate, vitamin B12 and homocysteine concentrations during fetal development may be associated with gestational age acceleration.

**Results:**

Up to 1346 newborns participating in the Generation R Study, a population-based prospective cohort study, had both cord blood DNA methylation data available and information on plasma folate, serum total and active B12 and plasma homocysteine concentrations, measured in early pregnancy and/or in cord blood. A subgroup of 380 newborns had mothers with optimal pregnancy dating based on a regular menstrual cycle and a known date of last menstrual period. For comparison, gestational age acceleration was calculated based the method of both Bohlin and Knight. In the total study population, which was more similar to Bohlin’s training population, one standard deviation score (SDS) higher maternal plasma homocysteine concentrations was nominally associated with positive gestational age acceleration [0.07 weeks, 95% confidence interval (CI) 0.02, 0.13] by Bohlin’s method. In the subgroup with pregnancy dating based on last menstrual period, the method that was also used in Knight’s training population, one SDS higher cord serum total and active B12 concentrations were nominally associated with negative gestational age acceleration [(− 0.16 weeks, 95% CI − 0.30, − 0.02) and (− 0.15 weeks, 95% CI − 0.29, − 0.01), respectively] by Knight’s method.

**Conclusions:**

We found some evidence to support associations of higher maternal plasma homocysteine concentrations with positive gestational age acceleration, suggesting faster epigenetic than clinical gestational aging. Cord serum vitamin B12 concentrations may be associated with negative gestational age acceleration, indicating slower epigenetic than clinical gestational aging. Future studies could examine whether altered fetal epigenetic aging underlies the associations of circulating homocysteine and vitamin B12 blood concentrations during fetal development with long-term health outcomes.

**Supplementary Information:**

The online version contains supplementary material available at 10.1186/s13148-021-01065-x.

## Introduction

Subtle differences in circulating maternal folate, vitamin B12 and homocysteine concentrations during pregnancy have been associated with offspring health outcomes [1–5]. DNA methylation may be a mechanism underlying these associations [6–9]. Folate, vitamin B12 and homocysteine are all part of the one-carbon metabolism, which is essential for cellular growth and differentiation and the biosynthesis, repair and methylation of DNA, among others [9]. Previously, a meta-analysis of two epigenome-wide association studies (EWAS) reported associations of maternal plasma folate concentrations with cord blood DNA methylation at 443 CpGs [10]. In addition, serum maternal vitamin B12 concentrations have been associated with local and global changes in newborn DNA methylation [11, 12]. A small study reported an inverse association between newborn homocysteine concentrations and LINE-1 methylation [13]. Active B12, or holotranscobalamin, is the biologically active fraction of vitamin B12, which is available to cells. The associations of active B12 concentrations with newborn DNA methylation have not been studied, even though it could possibly be a more reliable marker of an impaired vitamin B12 status, certainly in pregnancy [14].

Changes in DNA methylation have been associated with chronological aging [15]. Several “epigenetic clocks” have been developed as biomarkers of aging in adults [15–17]. Such clocks are based on DNA methylation levels at a limited number of CpGs (up to 500), specifically selected as predictors of chronological age [15]. Differences between DNA methylation-based estimates of age and chronological age are referred to as age acceleration. Positive age acceleration indicates an older DNA methylation-based age than chronological age. Negative age acceleration refers to a younger DNA methylation-based age than chronological age. In adults, positive age acceleration has been associated with all-cause mortality, as well as cancer and cardiovascular mortality [18, 19]. An intervention study among participants aged 65–75 years found that supplementation with folic acid and vitamin B12 reduced age acceleration, but only in a subgroup of women with normal activity of *MTHFR*, an enzyme involved in the one-carbon metabolism [20].

Recently, two epigenetic clocks for gestational age have been developed based on newborn DNA methylation data [21, 22]. Studies into associations of prenatal factors with gestational age acceleration have shown a mixed picture. Some prenatal exposures, such as maternal antenatal depression or insulin-treated gestational diabetes mellitus in a previous pregnancy, have been associated with negative gestational age acceleration, whereas others, such as maternal age and BMI, have been associated with positive age acceleration [23–26]. In addition, both positive and negative gestational age accelerations have been associated with various child health outcomes, such as growth, from birth onwards [22–24, 26, 27]. As such, the implications of gestational age acceleration still warrant further research [23–27].

In the current study, we hypothesized that higher plasma folate and serum B12 concentrations, and lower plasma homocysteine concentrations during fetal development are associated with negative gestational age acceleration. In a population-based study among 1346 mother–newborn pairs, we examined the associations of circulating folate, total and active B12 and homocysteine concentrations measured in early pregnancy and in cord blood with gestational age acceleration, estimated from cord blood DNA methylation data, using two epigenetic clocks for gestational age [21, 22].

## Results

### Subject characteristics

We included up to 1346 mother–newborn pairs with information on any exposure and cord blood DNA methylation data in the current study, which was embedded in the Generation R Study, a prospective cohort study from pregnancy onwards [28]. Additional file 1: Figure S1 shows a flowchart of the study population. Table 1 and Additional file 2: Table S1 show subject characteristics before and after imputation for covariates, respectively. We estimated DNA methylation gestational age from cord blood DNA methylation data, using both the epigenetic clock of Bohlin and Knight [21, 22]. The epigenetic clock of Bohlin estimates epigenetic gestational age based on DNA methylation levels of 96 CpGs from the HumanMethylation450 BeadChip selected trough Lasso regression (21). The epigenetic clock of Knight estimates gestational age based on methylation levels of 148 CpGs selected through elastic net regression, which are available on both the HumanMethylation27 and the HumanMethylation450 BeadChip [22]. Newborns had older clinically determined gestational age (median 40.3 weeks (95% range 36.7, 42.3)) than cord blood DNA methylation gestational age (Bohlin: median 39.4 weeks (95% range 37.0, 40.8); Knight: median 36.3 weeks (95% range 32.4, 39.1). This was reflected by the negative median values for raw gestational age acceleration, which were calculated as DNA methylation gestational age minus clinical gestational age. Spearman’s correlation between clinical and DNA methylation gestational age in the full group was higher for the epigenetic clock of Bohlin (r = 0.70), as compared to the epigenetic clock of Knight (r = 0.45) (Additional file 1: Figure S2). We observed the same among 380 newborns of mothers with optimal clinical pregnancy dating, based on a regular menstrual cycle and a known first date of last menstrual period (LMP), with Spearman’s correlations of r = 0.72 and r = 0.50 for Bohlin’s and Knight’s epigenetic clock, respectively [29]. Table 2 shows maternal plasma folate, serum total and active B12 and plasma homocysteine concentrations in early pregnancy and in cord blood. As expected in pregnancy, 30% of mothers had low serum total B12 concentrations according to the non-pregnant 95% reference interval [14]. Additional file 2: Table S2 shows a correlation matrix of all one-carbon metabolism markers. A non-response analysis showed that included mothers were more frequently older, higher educated, primiparous and non-smokers, on average had lower body mass indices (BMI) and higher circulating folate and vitamin B12 but similar homocysteine concentrations, as compared to non-included mothers (Additional file 2: Table S3). Included newborns were on average born heavier and at older clinical gestational ages, and more frequently had higher circulating folate but comparable vitamin B12 and homocysteine blood concentrations, as compared to non-included children.

**Table 1:** Maternal and child characteristics based on non-imputed data (n = 1346)

Maternal characteristics	
Age, year	31.4 (4.2)
Educational level	
No or primary	26 (2.0)
Secondary	439 (33.1)
Higher	826 (65.0)
Parity	
Nulliparous	817 (60.7)
Multiparous	528 (39.3)
Pre-pregnancy body mass index, kg/m^2^	22.3 (18.4, 33.7)
Smoking	
Non-smoker or smoked until pregnancy was known	1059 (85.7)
Smoked throughout pregnancy	176 (14.3)
Gestational age at blood sampling, week	12.8 (9.9, 16.9)
Newborn characteristics	
Gestational age at birth, week	40.3 (36.7, 42.3)
DNA methylation gestational age (Bohlin), week	39.4 (37.0, 40.8)
Raw gestational age acceleration (Bohlin), week	− 0.9 (− 2.7, 0.9)
DNA methylation gestational age (Knight), week	36.3 (32.4, 39.1)
Raw gestational age acceleration (Knight), week	− 3.7 (− 7.4, − 1.1)
Sex	
Boy	684 (50.8)
Girl	662 (49.2)
Birth weight, gram	3546 (510)

Values are mean (SD) or median (95% range) for continuous variables and numbers (%) for categorical variables. Missing data: DNA methylation gestational age and gestational age acceleration estimated by Bohlin’s epigenetic clock (n = 11), highest completed education (n = 19), parity (n = 1), pre-pregnancy BMI (n = 199), smoking (n = 111), gestational age at blood sampling in pregnancy (n = 223), birth weight (n = 1)

**Table 2:** Characteristics of circulating folate, vitamin B12 and homocysteine concentrations in early pregnancy and cord blood

	Early pregnancy	Cord blood
Plasma folate concentration, nmol/L	19.8 (6.6, 39.6)	21.2 (11.0, 38.4)
≥ 8 nmol/L	1041 (94.5)	1145 (99.9)
< 8 nmol/L	61 (5.5)	1 (0.1)
Serum total B12 concentration, pmol/L	178.0 (81.9, 428.5)	309.0 (128.0, 869.2)
≥ 145 pmol/L	726 (70.1)	1125 (95.7)
< 145 pmol/L	310 (29.9)	51 (4.3)
Serum active B12 concentration^a^, pmol/L	44.0 (21.0, 94.0)	87.0 (39.0, 128.0)
≥ 21 pmol/L	757 (97.8)	1124 (99.8)
< 21 pmol/L	17 (2.2)	2 (0.2)
Plasma homocysteine concentration, μmol/L	7.0 (4.7, 11.5)	9.1 (5.5, 16.4)
< 19 μmol/L	1082 (99.5)	1090 (99.0)
≥ 19 μmol/L	5 (0.5)	11 (1.0)

Values are based on non-imputed data and are median (95% range) for continuous variables and numbers (%) for categorical variables. We calculated standard deviation scores for all exposures to make them more comparable. Overall, 1346 mother–newborn pairs were included in one or more analyses. Not all pairs had information available on all exposure variables in either early pregnancy or cord blood. Missing data: maternal folate (n = 244), maternal total B12 (n = 310), maternal active B12 (n = 572), maternal homocysteine (n = 259), neonatal folate (n = 200), neonatal total B12 (n = 170), neonatal active B12 (n = 220), neonatal homocysteine (n = 245)

^a^Serum active B12 concentrations were measured in stored serum sampled in early pregnancy and cord blood. As not all participants had enough stored serum available, information on serum active B12 concentrations was available in fewer participants

### Folate, vitamin B12 and homocysteine blood concentrations and gestational age acceleration

We used linear regression models to examine the associations of early-pregnancy and cord blood plasma folate, serum total and active B12 and plasma homocysteine concentrations with gestational age acceleration. Both raw (DNA methylation gestational age minus clinical gestational age) and residual (residuals from regressing DNA methylation gestational age on clinical gestational age, see methods for details) gestational age acceleration were calculated, using two epigenetic clocks, of Bohlin and of Knight [21, 22]. In the full study population, using Bohlin’s method, one standard deviation score (SDS) higher maternal early-pregnancy plasma homocysteine concentrations was nominally associated with gestational age acceleration (0.07 weeks, 95% confidence interval (CI): 0.02, 0.13) in the main model, which was adjusted for maternal age, education, pre-pregnancy BMI, parity and smoking, child sex, batch, estimated cell types and gestational age at blood sampling (Table 3). This association at nominal significance did remain if we applied a Bonferroni correction, adjusting for four exposures. In “mediator” models with additional adjustment for either birth weight, folate or vitamin B12, these associations had comparable effect estimates, but attenuated into non-significance (Additional file 2: Table S4). None of the exposures were associated with gestational age acceleration using Knight’s method (Table 3, all *P* values ≥ 0.05). For both epigenetic clocks, the associations were similar without correction for cell type proportions (Additional file 2: Table S5). The results of the crude (adjusted for batch, sex and also for gestational age at blood sampling in early pregnancy models) and basic (additionally adjusted for cell type proportions) models are shown in Additional file 2: Tables S6 and S7. The results of the main models comparing low versus normal maternal serum total B12 concentrations, based on the non-pregnant 95% reference interval, are provided in Additional file 2: Table S8. Additional file 2: Table S9 displays results of sensitivity analyses on cord serum active B12 concentrations, after exclusion of 189 newborns with concentrations recorded as the upper limit of measurement of the used immuno-assay. Effect estimates of the associations were largely similar to those from the primary models. There were no indications for non-linear associations between any exposure and both measures of gestational age acceleration, using both epigenetic clocks (data not shown).

**Table 3:** Associations of circulating folate, vitamin B12 and homocysteine concentrations with gestational age acceleration (full population)

	Bohlin				Knight			
	Raw acceleration^a^		Residual acceleration^b^		Raw acceleration^a^		Residual acceleration^b^	
	Difference (95% CI) in weeks	*P* value	Difference (95% CI)	*P* value	Difference (95% CI) in weeks	*P* value	Difference (95% CI)	*P* value
Early pregnancy								
Folate, SDS	0.02 (− 0.03, 0.08)	0.44	0.00 (− 0.03, 0.04)	0.80	− 0.01 (− 0.10, 0.08)	0.87	− 0.02 (− 0.10, 0.06)	0.62
Total B12, SDS	− 0.01 (− 0.07, 0.05)	0.72	0.00 (− 0.03, 0.04)	0.80	− 0.04 (− 0.13, 0.05)	0.38	− 0.03 (− 0.10, 0.05)	0.50
Active B12, SDS	− 0.03 (− 0.10. 0.03)	0.31	0.00 (− 0.04. 0.04)	0.98	− 0.05 (− 0.15, 0.05)	0.33	− 0.03 (− 0.11, 0.06)	0.59
Homocysteine, SDS	0.07 (0.02, 0.13)	0.01*	0.01 (− 0.02, 0.04)	0.45	0.07 (− 0.01, 0.16)	0.09	0.03 (− 0.05, 0.10)	0.54
Cord blood								
Folate, SDS	0.02 (− 0.03, 0.08)	0.41	0.01 (− 0.02, 0.04)	0.56	0.04 (− 0.04, 0.13)	0.32	0.03 (− 0.04, 0.11)	0.41
Total B12, SDS	− 0.03 (− 0.09, 0.02)	0.25	0.02 (− 0.01, 0.05)	0.18	− 0.03 (− 0.12, 0.05)	0.42	0.01 (− 0.06, 0.08)	0.73
Active B12, SDS	0.03 (− 0.03, 0.08)	0.34	0.02 (− 0.01. 0.05)	0.24	0.01 (− 0.07, 0.10)	0.76	0.01 (− 0.07, 0.08)	0.86
Homocysteine, SDS	− 0.00 (− 0.06, 0.05)	0.93	− 0.01 (− 0.05. 0.02)	0.40	0.03 (− 0.05, 0.22)	0.49	0.02 (− 0.06, 0.09)	0.62

*This association at nominal significance did remain if we applied a Bonferroni correction, adjusting for four exposures (0.05/4)

The full study population included n = 1346 mother–newborn pairs for the analysis using Knight’s epigenetic clock and n = 1335 mother–newborn pairs for the analysis using Bohlin’s epigenetic clock. Values represent regression coefficients (95% confidence interval) and reflect the difference in raw and residual gestational age acceleration at birth per increase of 1 standard deviation score in exposure variable. Results are based on the main models, which were adjusted for maternal age, education, pre-pregnancy BMI, parity and smoking, child sex, batch effects (by including plate number), cell types, and additionally for gestational age at blood sampling in early pregnancy models. Folate and homocysteine concentrations were measured in plasma and total and active B12 concentrations were measured in serum

*CI* confidence interval, *SDS* standard deviation score

^a^Raw gestational age acceleration (in weeks) was obtained by subtracting the clinical estimate of gestational age from DNA methylation gestational age

^b^Residual gestational age acceleration (no unit) was calculated from the residuals from a regression model of DNA methylation gestational age on clinical gestational age

When we restricted the analyses to up to 380 newborns of mothers with optimal pregnancy dating based on a regular menstrual cycle and a known first day of LMP, using Knight’s method, one SDS (188.1 pmol/L) increase in cord serum total B12 concentrations was associated with − 0.16 weeks (95% CI − 0.30, − 0.02) raw gestational age acceleration at nominal significance (Table 4). Also, one SDS (27.9 pmol/L) increase in cord serum active B12 concentrations was associated with − 0.15 weeks (95% CI − 0.29, − 0.01) raw gestational age acceleration. The associations did not remain when adjusting for multiple testing. Neither of these associations changed substantially in the “mediator” models (Additional file 2: Table S10). In the subgroup, none of the exposures were associated with gestational age acceleration using Bohlin’s method (Table 4, all *P* values ≥ 0.05). We did not stratify the folate analyses on newborn rs1801133 (*MTHFR*) genotype, nor the vitamin B12 analyses on rs3742801 (*ABCD4*) genotype, since the interaction terms were not significant (*P* values ≥ 0.05).

**Table 4:** Associations of circulating folate, vitamin B12 and homocysteine concentrations with gestational age acceleration (subgroup analysis)

	Bohlin				Knight			
	Raw acceleration^a^		Residual acceleration^b^		Raw acceleration^a^		Residual acceleration^b^	
	Difference (95% CI) in weeks	*P* value	Difference (95% CI)	*P* value	Difference (95% CI) in weeks	*P* value	Difference (95% CI)	*P* value
Early pregnancy								
Folate, SDS	− 0.01 (− 0.11, 0.10)	0.89	0.02 (− 0.04, 0.08)	0.50	− 0.01 (− 0.15, 0.13)	0.88	0.02 (− 0.10, 0.14)	0.75
Total B12, SDS	0.02 (− 0.08, 0.12)	0.71	0.03 (− 0.03, 0.09)	0.36	− 0.03 (− 0.17, 0.12)	0.71	− 0.01 (− 0.14, 0.11)	0.82
Active B12, SDS	− 0.03 (− 0.14, 0.08)	0.60	0.01 (− 0.05, 0.08)	0.72	− 0.07 (− 0.22, 0.08)	0.36	− 0.04 (− 0.17, 0.10)	0.60
Homocysteine, SDS	0.04 (− 0.07, 0.16)	0.47	− 0.04 (− 0.11, 0.03)	0.25	− 0.02 (− 0.16, 0.12)	0.79	− 0.08 (− 0.20, 0.04)	0.20
Cord blood								
Folate, SDS	− 0.09 (− 0.19, 0.02)	0.11	− 0.04 (− 0.10, 0.03	0.24	0.05 (− 0.09, 0.18)	0.52	0.08 (− 0.04, 0.20)	0.18
Total B12, SDS	− 0.10 (− 0.20, 0.00)	0.06	− 0.03 (− 0.09, 0.03)	0.37	− 0.16 (− 0.30, − 0.02)	0.02*	− 0.10 (− 0.22, 0.02)	0.12
Active B12, SDS	− 0.11 (− 0.21, 0.00)	0.06	− 0.05 (− 0.11, 0.01)	0.12	− 0.15 (− 0.29, − 0.01)	0.04*	− 0.10 (− 0.23, 0.02)	0.10
Homocysteine, SDS	− 0.00 (− 0.10, 0.09)	0.93	− 0.01 (− 0.06. 0.05)	0.78	− 0.00 (− 0.14, 0.14)	0.97	− 0.01 (− 0.13, 0.12)	0.91

*This association at nominal significance did remain if we applied a Bonferroni correction, adjusting for four exposures (0.05/4)

This analysis included 380 newborns of mothers with optimal pregnancy dating based on a regular menstrual cycle and gestational age determined by last menstrual period. For the analysis using Bohlin’s epigenetic clock we included 378 of these newborns, after excluding 2 newborns with missing data for some CpGs required for the DNA methylation gestational age calculation. Values represent regression coefficients (95% confidence interval) and reflect the difference in raw and residual gestational age acceleration at birth per increase of 1 standard deviation score in exposure variable. Results are based on the main models, which were adjusted for maternal age, education, pre-pregnancy BMI, parity and smoking, child sex, batch effects (by including plate number), cell types, and additionally for gestational age at blood sampling in early pregnancy models. Folate and homocysteine concentrations were measured in plasma and total and active B12 concentrations were measured in serum

*CI* confidence interval, *SDS* standard deviation score

^a^Raw gestational age acceleration (in weeks) was obtained by subtracting the clinical estimate of gestational age from DNA methylation gestational age

^b^Residual gestational age acceleration (no unit) was calculated from the residuals from a regression model of DNA methylation gestational age on clinical gestational age

## Discussion

This study found some evidence that higher maternal plasma homocysteine concentrations are associated with positive age acceleration, referring to faster epigenetic as compared to clinical gestational aging. In addition, higher cord serum total and active B12 concentrations may be associated with negative gestational age acceleration, indicating slower epigenetic aging as compared to clinical gestational aging.

Folate, vitamin B12 and homocysteine interact in the one-carbon metabolism, which is involved in the donation of methyl groups for DNA methylation [9]. Previous studies have reported associations of these micronutrients in blood with newborn DNA methylation [10–13]. Altered epigenetic aging in response to the environment in utero, resulting in gestational age acceleration, might be an underlying mechanism for the associations of circulating folate, vitamin B12 and homocysteine concentrations during pregnancy and health outcomes in children [2, 3, 5, 9, 20]. We hypothesized that subtle differences in circulating folate, vitamin B12 and homocysteine concentrations during fetal development are associated with gestational age acceleration.

In the full cohort, using Bohlin’s epigenetic clock, higher maternal plasma homocysteine concentrations were associated with positive raw gestational age acceleration. In a subgroup of newborns of mothers with optimal pregnancy dating, using Knight’s epigenetic clock, higher cord serum vitamin B12 concentrations were associated with negative raw gestational age acceleration. These findings are in line with our hypothesis. Previous work from birth cohorts reported associations of lower circulating homocysteine concentrations but higher circulating vitamin B12 concentrations during fetal development with beneficial health outcomes from birth onwards [2, 3, 5]. Changes in epigenetic gestational age may be an underlying mechanism. In adults, positive age acceleration is associated with worse outcomes [18, 19]. Folic acid and vitamin B12 supplementation have been associated with reduced age acceleration in a specific subgroup of elderly women with *MTHFR 677CC* genotype [20]. However, it is not evident whether findings in adults can directly be translated to gestational age acceleration in newborns. Previously, various exposures during pregnancy have been associated with both positive and negative gestational age acceleration. Insulin-treated gestational diabetes mellitus in a previous pregnancy, maternal Sjögren’s syndrome and antenatal depression have all been associated with negative gestational age acceleration [24, 26]. In contrast, maternal age [24] and BMI [23], pre-eclampsia [24], medication use and complications or interventions in pregnancy [24] have been associated with positive gestational age acceleration [22–26], as has vitamin D supplementation [25]. Birth size has previously been associated with both positive and negative gestational age acceleration [23, 24, 27]. Also, one study reported that associations of gestational age acceleration with child weight and length at various ages were not consistent in direction [27]. Therefore, rather than the linear interpretation in adults, a too strong acceleration, be it positively or negatively, may represent a suboptimal situation in newborns.

Our findings differed slightly between the two epigenetic clocks. However, the effect size of the association of higher maternal plasma homocysteine concentrations with positive gestational age acceleration was the same for both Bohlin’s and Knight’s epigenetic clock. In contrast, for the associations of higher cord serum vitamin B12 concentrations with negative raw gestational age acceleration, observed for Knight’s epigenetic clock in the subgroup with optimal pregnancy dating, the effect estimates were 30% smaller when using Bohlin’s epigenetic clock. A recent study examined associations of vitamin D supplementation with gestational age acceleration and found similar results using both Bohlin’s and Knight’s epigenetic clock [25]. However, the authors also reported differences between the two epigenetic clocks and their associations with exposures during pregnancy. Only gestational age acceleration based on Bohlin’s epigenetic clock was associated with higher maternal BMI and higher birth weight, whereas only gestational age acceleration based on Knight’s epigenetic clock was associated with child sex. Differences in findings, depending on which epigenetic clock is used, could result from the resemblance of the study population under investigation with the training datasets used to develop either Bohlin’s or Knight’s epigenetic clock. In our full population, newborns were comparable to the newborns in which the Bohlin clock was developed, being from European ethnicity and with similar mean clinical gestational age of 39.9 weeks [21, 30]. In contrast, the training dataset used to develop Knight’s epigenetic clock was of mixed ethnic background and characterized by a younger mean clinical gestational age of 36.9 weeks, which was estimated based on LMP, similar to the included newborns in our subgroup analysis [22, 30]. In line with this, in our full population, the correlation between clinical and DNA methylation gestational age was higher when based on Bohlin’s epigenetic clock, as compared to Knight’s epigenetic clock. This suggests that epigenetic age estimated by Bohlin’s epigenetic clock may be more accurate than epigenetic age estimated by Knight’s epigenetic clock in our population. In the subgroup with optimal pregnancy dating based on LMP, the correlation with Knight’s epigenetic clock increased slightly, potentially because clinical gestational age was estimated similarly. An alternative explanation for differences between findings based on the used epigenetic clock may be that Bohlin’s and Knight’s epigenetic clock contain different CpGs, which may be indicative of different aspects of accelerated aging. Only one CpG (cg05365729) overlaps between the epigenetic clocks of Bohlin and Knight. We did not observe any associations for plasma folate concentrations. None of the differentially methylated CpGs in the previously published meta-analysis of EWASs on maternal folate concentrations were included in either Bohlin’s or Knight’s epigenetic clock [10, 22]. Variation in plasma folate concentrations may thus not be reflected in epigenetic accelerated gestational aging. A different explanation could be that folate may be associated with differential DNA methylation at specific CpGs, but not with DNA methylation changes relevant for accelerated aging. Both our findings for circulating homocysteine and vitamin B12 concentrations, and the null results for circulating folate concentrations, require further replication.

We found no associations of cord serum vitamin B12 with gestational age acceleration in the full population, in which mothers with less optimal pregnancy dating were included. Measurement errors in fetal ultrasound imaging may yield inaccurate estimates of clinical gestational age. Such errors subsequently have consequences for the accuracy of estimating gestational age acceleration. In addition, pregnancy dating based on fetal ultrasound reduces biological variation in early fetal growth, which may result from environmental factors such as nutrition, including folate, vitamin B12 and homocysteine [29]. Thus, we consider the pregnancy dating in the subgroup to be more optimal than in the full group, which may have led to increased precision in the gestational age acceleration outcomes. However, the observed association between maternal plasma homocysteine concentrations and gestational age acceleration estimated by Bohlin’s epigenetic clock in the full population was not found in the subgroup, even though the correlation between clinical and Bohlin’s estimation of DNA methylation gestational age increased. Whether this was due to lower power, which was not outweighed by increased precision in the clinical gestational age measurement, needs to be confirmed in further studies.

We observed associations of plasma homocysteine and serum vitamin B12 concentrations with the raw, but not residual estimate of gestational age acceleration. In contrast, the previously described study on vitamin D supplementation during pregnancy and associations with gestational age acceleration reported significant associations only for the residual estimate [25]. Future studies should examine whether the current findings for plasma homocysteine and serum vitamin B12 concentrations with raw gestational age acceleration represent true associations, as raw gestational age might be confounded by clinical gestational age. Still, calculating both raw and residual gestational age acceleration seems justifiable as both measures might serve different purposes [24]. Consequences of gestational age acceleration may depend on clinical gestational age. For example, raw gestational age acceleration of one week may have different implications for a neonate born at 40 weeks gestational age, as compared to a premature born at 30 weeks. The raw estimate may be more useful and clinically relevant at individual level. In contrast, residual age acceleration may be appropriate for testing hypotheses at population level, as it is uncorrelated with clinical gestational age [24].

We observed associations of maternal plasma homocysteine concentrations in pregnancy, but not in cord blood, with gestational age acceleration. In contrast, in the subgroup with optimal pregnancy dating, we observed nominal associations of cord blood but not early-pregnancy serum vitamin B12 concentrations with gestational age acceleration. If cord blood is a proxy for the third trimester, our findings could indicate that the first trimester is the critical period for associations of circulating homocysteine concentrations with gestational age acceleration, whereas the third trimester is the critical period for associations of circulating vitamin B12 concentrations with gestational age acceleration. However, this hypothesis needs further study.

### Strengths and limitations

This study is embedded in a large observational cohort study. For the measurement of DNA methylation, we selected an ethnically homogeneous European-ancestry subgroup of newborns. This may limit the generalizability of our findings to other ethnicities. The availability of serum active B12 measurements, which potentially better reflects circulating vitamin B12 status than total B12, is a major strength of our study. Although this cohort has a relatively large set of DNA methylation data available, it could be that we did not have not enough power to find more true associations. Future larger (meta-)analyses could shed more light on this. Our study included relatively few participants with low circulating folate and active B12 concentrations, or high homocysteine concentrations, although specific clinical cutoff values for pregnant women do not exist. It may be that more extreme variations in folate, vitamin B12 and homocysteine blood concentrations are associated with gestational age acceleration. Blood samples were stored at room temperature for up to 3 h, which may have led to time-dependent increases in homocysteine concentrations due to a continuous production and release of homocysteine from blood cells [31, 32]. This may have limited our capacity to detect significant associations between homocysteine and all outcomes.

## Conclusions

We found some indication that maternal plasma homocysteine concentrations may be associated with positive gestational age acceleration. In addition, cord serum vitamin B12 concentrations may be associated with negative gestational age acceleration. This could imply that altered epigenetic aging in response to subtle differences in homocysteine and vitamin B12 concentrations might be an underlying mechanism for the associations of these micronutrients during fetal development and child health outcomes. Future studies should replicate our analyses among larger samples of mothers with optimal pregnancy dating and newborns from different underlying populations.

## Methods

### Participants

This study was embedded in the Generation R Study, a population-based prospective cohort study from fetal life onwards in Rotterdam, the Netherlands [28]. The Medical Ethical Committee of Erasmus MC, University Medical Center Rotterdam, approved the study (MEC 198.782/2001/31). Pregnant women with an expected delivery date between April 2002 and January 2006 living in Rotterdam were eligible to participate, and written informed consent was obtained from all participants. We measured folate, total and active B12 and homocysteine concentrations in maternal blood drawn in early pregnancy and in cord blood. In 1396 of the 9901 live-born newborns participating in the Generation R Study, we measured genome-wide DNA methylation in cord blood. This subgroup was selected from the total study population as a relatively homogeneous, European-ancestry subgroup. In the current study, we included mother–newborn pairs who met the following criteria: information on gestational age at birth and neonatal DNA methylation data available and at least one measurement of circulating folate, total or active B12 or homocysteine concentrations in either pregnancy or cord blood. Among these were 15 mothers with two (non-twin) children. Per mother, we included only one child by first selecting based on completeness of exposure data, and if equal, based on completeness of covariates.

### Maternal and neonatal folate, vitamin B12 and homocysteine blood concentrations

Maternal venous blood samples were drawn in early pregnancy (gestational age ≤ 17 weeks) in a dedicated research center under non-fasting conditions, and cord blood samples were taken immediately after delivery [2]. After collection, blood samples were stored at room temperature for a maximum of 3 h, before being transported to the regional laboratory for processing and storage at − 80 °C. Folate and homocysteine concentrations were measured in EDTA plasma, and total and active B12 concentrations were measured in serum in the Department of Clinical Chemistry at Erasmus MC, University Medical Centre Rotterdam. After thawing, folate, vitamin B12 and homocysteine concentrations were analyzed using an immune-electro-chemo-luminescence assay on the Architect System [2]. Concentrations below or above the measuring ranges of this assay for folate (1.8–45.3 nmol/l), total B12 (44–1476 pmol/l), active B12 (5–128 pmol/l) and homocysteine (1–50 µmol/l) could not be quantified and were recorded as either the lower or the upper limit of the measuring range of the assay (n = 9 and n = 8 for maternal and neonatal folate, respectively; n = 1 and n = 2 for maternal and neonatal total B12, respectively; n = 4 and n = 189 for maternal and neonatal active B12, respectively; n = 0 for both maternal and neonatal homocysteine). We also dichotomized maternal and cord blood folate (≥ 8 and < 8 nmol/L, respectively), total B12 (≥ 145 and < 145 pmol/L, respectively), active B12 (≥ 21 and < 21 pmol/L, respectively) and homocysteine (< 19 and ≥ 19 μmol/L, respectively) concentrations according to the 95% reference interval for healthy adults [2, 9, 33]. We only explored whether low maternal serum total B12 status showed different associations with gestational age acceleration, as compared to normal maternal serum total B12 status. For the other exposures, the percentage of mothers or newborns with abnormal values was deemed too small for meaningful analyses.

### DNA methylation data

We used the salting-out method to extract DNA from cord blood samples. Five-hundred nanograms of DNA were bisulfite converted using the EZ-96 DNA Methylation kit (Shallow) (Zymo Research Corporation, Irvine, USA). Samples were processed with the Illumina Infinium HumanMethylation450 BeadChip (Illumina Inc., San Diego, USA). Quality control and normalization were performed using the CPACOR workflow [34]. Probes with a detection *p* ≥ 1E−16 were set to missing. Intensity values were quantile normalized. We removed arrays with technical problems, a call rate ≤ 95%, or a mismatch between the expected sex of participant and sex determined by chromosome X and Y probe intensities. Probes on the sex chromosomes were removed before the analyses. We used untransformed beta-values as measures of DNA methylation. The final DNA methylation dataset contained information on 458,563 CpGs.

### Gestational age estimation and gestational age acceleration

Participating pregnant mothers were seen in the first trimester of pregnancy for fetal ultrasound at our research center [35]. During this visit, gestational age was established. If mothers had a known and reliable first day of the LMP, and a regular menstrual cycle of 28 ± 4 days, the clinical estimate of gestational age was based on LMP [35]. If mothers did not know the exact date of their LMP, or had an irregular menstrual cycle, we established gestational age by ultrasound examination, which does not take into account variation in early fetal growth [35]. Measurement error in gestational age acceleration may occur if either the clinical or the DNA methylation gestational age estimate is inaccurate. Therefore, we selected 380 newborns of mothers with optimal pregnancy dating based on LMP for sensitivity analyses. Gestational age at birth was assessed from midwife or obstetric records.

We calculated DNA methylation gestational age using two different epigenetic clocks, to test the robustness of our results. We used the GA prediction package version 1.16.0 in R 3.6.1 to calculate DNA methylation gestational age based on the epigenetic clock developed by Bohlin [21, 36]. Bohlin estimates epigenetic gestational age based on DNA methylation levels of 96 CpGs from the HumanMethylation450 BeadChip selected trough Lasso regression (21). None of the 96 required CpGs were missing in our dataset, but we excluded newborns with missing values for one or more of the required CpGs (total study population: n = 11; subgroup with optimal pregnancy dating: n = 2). We used the methylclock package 0.5.0 in R 3.6.1 to calculate DNA methylation gestational age based on the epigenetic clock developed by Knight [22, 37, 38]. Knight’s epigenetic clock estimates gestational age based on methylation levels of 148 CpGs selected through elastic net regression, which are available on both the HumanMethylation27 and the HumanMethylation450 BeadChip [22, 24]. None of the 148 required CpGs were missing in our dataset. We calculated both raw and residual gestational age acceleration based on the epigenetic clocks of Bohlin and Knight and analyzed both measures of acceleration, in line with previous research [24, 25, 37]. Raw gestational age acceleration (in weeks) was obtained by subtracting the clinical estimate of gestational age from DNA methylation gestational age. This measure is straightforward, but does not take into account the potential confounding effect of clinical gestational age on DNA methylation gestational age, as variance is shared between clinical and DNA methylation gestational age [24]. Residual gestational age acceleration was calculated as the residuals from a regression model of DNA methylation gestational age on clinical gestational age [37]. By definition, this measure is uncorrelated with clinical gestational age [24]. Positive gestational age acceleration was defined as older DNA methylation gestational age than clinical gestational age; negative gestational age acceleration was defined as younger DNA methylation gestational age than clinical gestational age.

### Covariates

We selected potential covariates based on previous literature. Maternal covariates included age at conception, educational level, pre-pregnancy BMI, parity, smoking during pregnancy and the early-pregnancy analyses also gestational age at blood sampling. The latter was included because physiologically concentrations of all exposures decline during pregnancy [9]. In addition, we included child sex as a covariate. We obtained information on maternal covariates from questionnaires sent out in each trimester of pregnancy. Information on child sex and birth weight, for which we calculated sex- and gestational age-dependent standard deviation scores, was obtained from midwife and hospital records [39]. We considered birth weight as a potential mediator. We used the “Salas” reference set for the estimation of cell type proportion in the “FlowSorted.CordBlood.Combined.450 K” Bioconductor package [40]. This reference set includes the following cell types: CD8+ T cells, CD4+ T cells, natural killer cells, B cells, monocytes, granulocytes, nucleated red blood cells. We tested for an interaction between plasma folate concentrations and newborn rs1801133 (*MTHFR*) genotype*,* and between serum vitamin B12 concentrations and rs3742801 (*ABCD4*) genotype [41]. These SNPs were selected because of their expected roles in fetal folate and vitamin B12 metabolism, respectively [42].

### Statistical analysis

First, we performed a non-response analysis, using Student’s t-tests, Mann–Whitney tests and Chi-square tests. We compared characteristics of newborns included in the analyses, to not included newborns, which were not selected for cord blood DNA methylation measurement, had no information on any exposure or had a sibling that was included in the analyses. Second, we calculated Spearman’s correlation coefficients between clinical and DNA methylation gestational age and between maternal and neonatal exposures. To compare effect estimates, we analyzed folate, total and active B12 and homocysteine blood concentrations continuously per SDS. We examined the associations of maternal and neonatal folate, total and active B12 and homocysteine blood concentrations with raw and residual gestational age acceleration, calculated based on Bohlin’s and Knight’s method, using linear regression models in R 3.6.1 [38]. Crude models were adjusted for batch effects by including plate number, for sex and also for gestational age at blood sampling in the early-pregnancy models. Basic models were additionally adjusted for estimated cell types. Main models were additionally adjusted for maternal age, education, pre-pregnancy BMI, parity and smoking. To examine the impact of variation in cell type proportions, the main model was also run without cell type adjustment (reduced main model), in line with previous research [23]. We also analyzed the associations of normal versus low (< 145 pmol/L) maternal total B12 status with gestational age acceleration. For the other exposures, the percentage of participants with concentrations outside the 95% reference interval was very low, and therefore similar analyses were not performed. We further assessed whether our results for active B12 changed after excluding the 189 newborns with serum active B12 concentrations corresponding to the upper limit of the analytic range of the immuno-assay. For other exposures, we did not perform such sensitivity analyses, because less than ten participants had blood concentrations corresponding to the limit of the analytic range of the immuno-assay. Only in case of significant associations in the main model, we ran mediator models, additionally adjusting for birth weight (birth weight model), folate (folate model), vitamin B12 (total B12 model and active B12 model) and homocysteine (homocysteine model), to explore whether any associations were explained by these covariates. Finally, to examine potential nonlinearity of the associations, we studied both the maternal and newborn exposures in quintiles, with the third quintile as the reference category. We used multiple imputation for covariates with missing values, using the Markov chain Monte Carlo method. We created five datasets and analyzed these together [43]. We performed all statistical analyses using the Statistical Package for the Social Sciences version 25.0 (SPSS IBM, Chicago, Illinois, USA). We considered *P* values < 0.05 statistically significant.

## Supplementary Information

**Additional file 1: Figure S1**. Flowchart of the study population. **Figure S2**. Correlation between clinical and DNA methylation gestational age.

**Additional file 2: Table S1**. Maternal and child characteristics based on imputed data (n = 1346). **Table S2**. Correlation matrix of circulating folate, vitamin B12 and homocysteine concentrations. **Table S3**. Non-response analysis. **Table S4**. Associations of maternal plasma homocysteine concentrations with raw gestational age acceleration estimated by Bohlin’s epigenetic clock (mediator models). **Table S5**. Associations of circulating folate, vitamin B12 and homocysteine concentrations with gestational age acceleration (reduced main models). **Table S6**. Associations of circulating folate, vitamin B12 and homocysteine concentrations with gestational age acceleration (crude models). **Table S7**. Associations of circulating folate, vitamin B12 and homocysteine concentrations with gestational age acceleration (basic models). **Table S8**. Associations of low versus normal early-pregnancy serum total B12 concentrations with gestational age acceleration. **Table S9**. Associations of cord serum active B12 concentrations with gestational age acceleration (sensitivity analysis). **Table S10**. Associations of cord serum vitamin B12 concentrations with gestational age acceleration estimated by Knight’s epigenetic clock (subgroup analysis, mediator models)

## Data Availability

The datasets generated and/or analyzed during the current study are not publicly available due to privacy restrictions, but are available from the corresponding author on reasonable request, subject to the Generation R Study executive data access procedures.

## Abbreviations

BMI: Body mass index
CI: Confidence interval
CpG: Cytosine-guanine dinucleotide
CpGs: CpG sites
EWAS: Epigenome-wide association study
SD: Standard deviation
SDS: Standard deviation score
