# Supplementary material for: Associations of circulating folate, vitamin B12 and homocysteine concentrations in early pregnancy and cord blood with epigenetic gestational age: the Generation R Study
Source: Clin Epigenetics. 2021 Apr 29;13:95. doi: 10.1186/s13148-021-01065-x (PMC8082638; doi:10.1186/s13148-021-01065-x)
Supplement: Supplementary file 1 — Additional file 1: Figure S1. Flowchart of the study population. Figure S2. Correlation between clinical and DNA methylation gestational age. [file 13148_2021_1065_MOESM1_ESM.pdf]

Associations of circulating folate, vitamin B12 and homocysteine concentrations in early pregnancy and cord blood with epigenetic gestational age: the Generation R Study

Additional file 1

Giulietta S. Monasso[1,2], Leanne K. Küpers[1,2,3], Vincent W.V. Jaddoe[1,2], Sandra G. Heil[4], Janine F. Felix[1,2]

1. The Generation R Study Group, Erasmus MC, University Medical Center Rotterdam, Rotterdam, the Netherlands;
2. Department of Pediatrics, Erasmus MC, University Medical Center Rotterdam, Rotterdam, the Netherlands.
3. Division of Human Nutrition and Health, Wageningen University, Wageningen, The Netherlands
4. Department of Clinical Chemistry, Erasmus MC, University Medical Center Rotterdam, Rotterdam, the Netherlands.

Content

- **Figures: 2**

Corresponding author: Janine F. Felix, MD PhD, Generation R Study Group (Na-2918), Erasmus MC, University Medical Center Rotterdam, Rotterdam, the Netherlands. Phone: +31 10 7043405, fax: +31 10 70 44645, email: j.felix@erasmusmc.nl

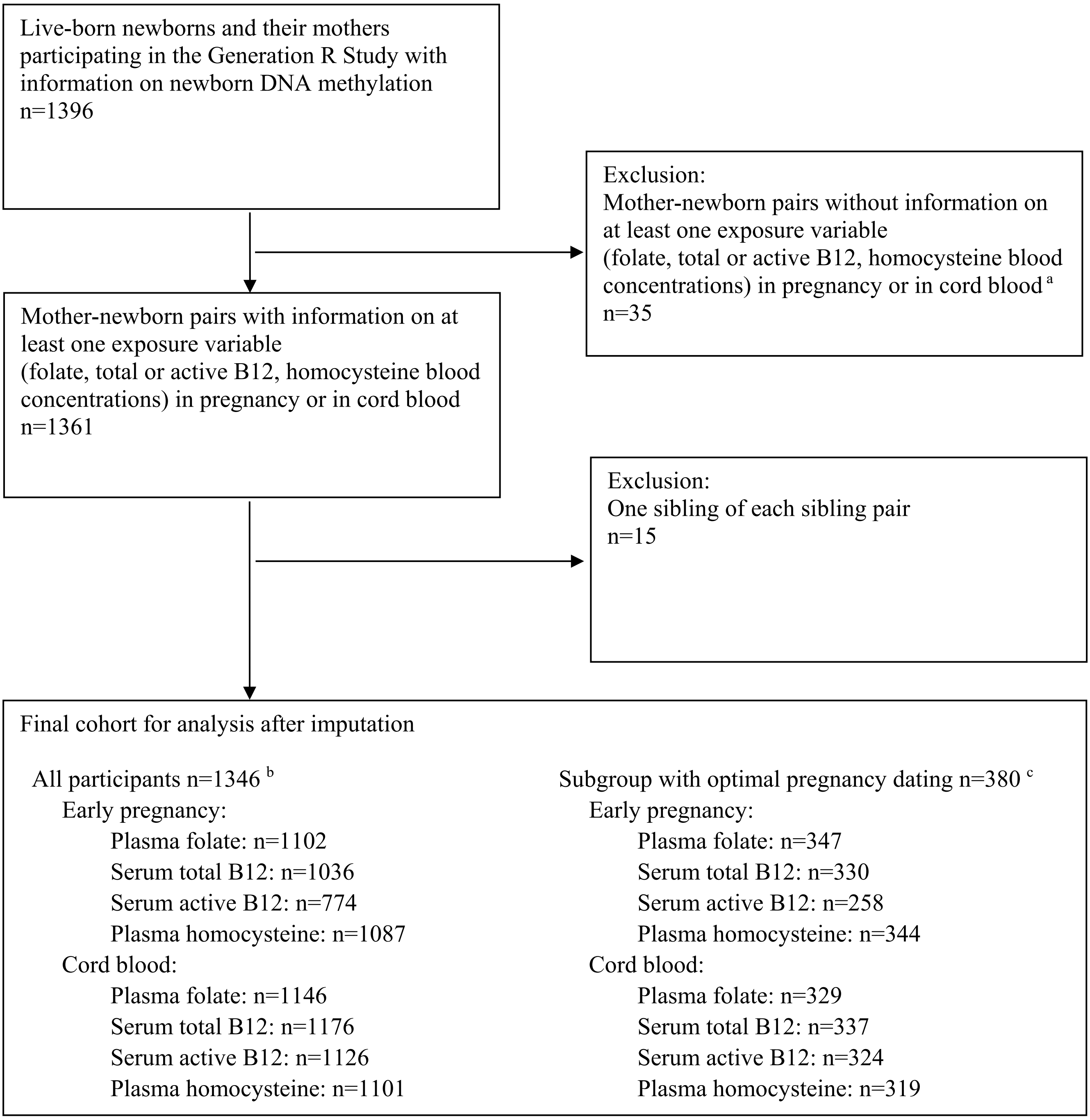

Figure S1. Flow chart of the study population

a The non-response analysis compared the newborns who were included in the analyses to those who participated in Generation R at birth but were not included (n=8555), because either no DNA methylation was measured (n=8505), or information on all exposures was missing (n=35), or their sibling was included in the analyses (n=15).

b For the analyses based on Bohlin's epigenetic clock, we excluded 11 newborns with missing values for some of the required CpGs, leaving n=1335 children for analysis.

c Based on a regular menstrual cycle of 28 ± 4 days and a known date of last menstrual period. For the analyses based on Bohlin's epigenetic clock, we excluded 2 newborns with missing values for some of the required CpGs, leaving n=378 children for analysis.

Supplemental Figure 2. Correlation between clinical and DNA methylation gestational age

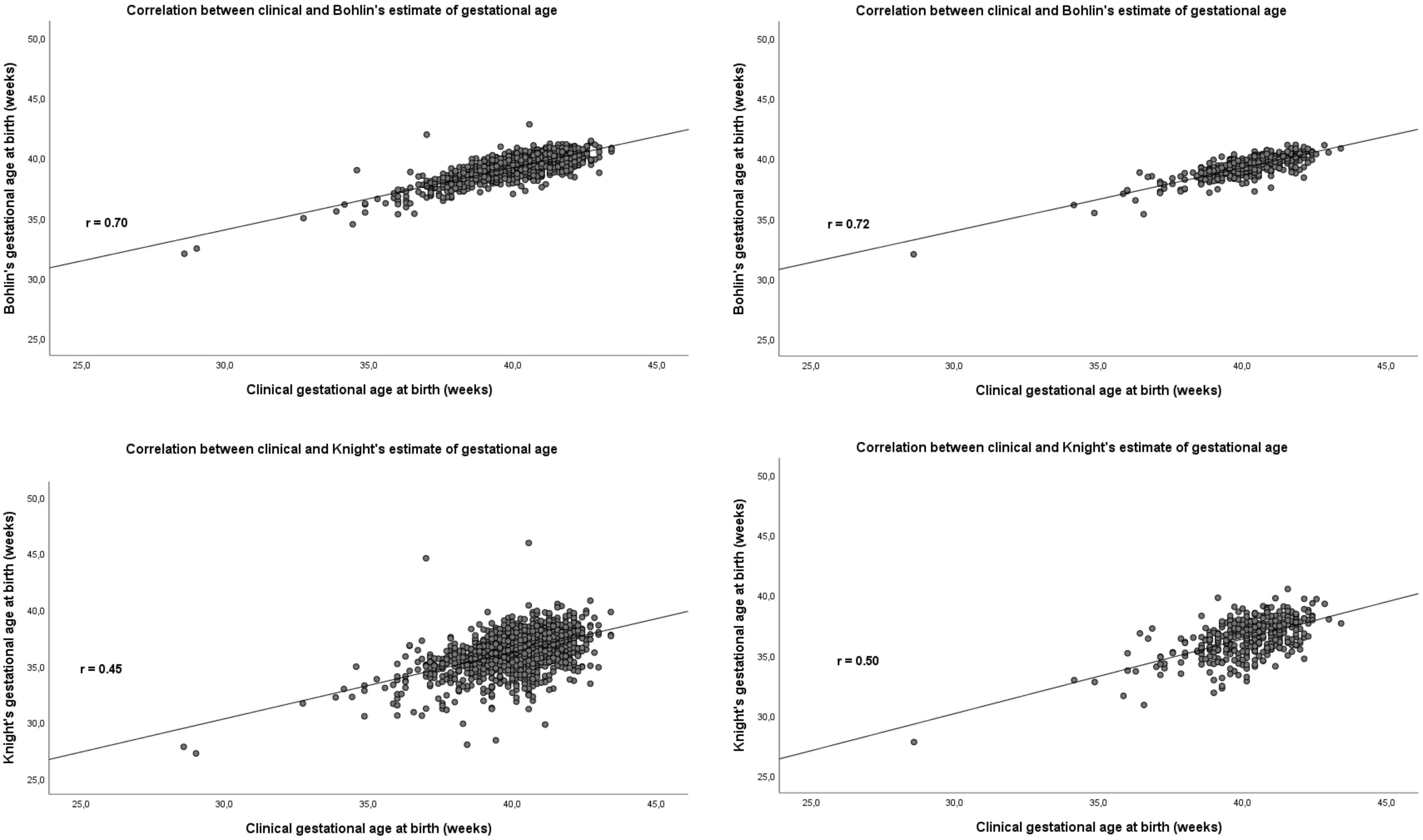

Panels show Spearman's correlation between gestational age at birth as determined by clinical methods (x-axes) and DNA methylation gestational age estimated by Bohlin's or Knight's epigenetic clock (y-axes) for gestational age.
Upper left panel: The correlation between clinical and DNA methylation gestational age calculated among all n=1335 newborns included in the analyses using Bohlin's clock.
Upper right panel: The correlation between clinical and DNA methylation gestational age using Bohlin's clock calculated among n=378 newborns of mothers with optimal pregnancy dating based on having a regular menstrual cycle and a known first date of last menstrual period.
Lower left panel: The correlation between clinical and DNA methylation gestational age calculated among all n=1346 newborns included in the analyses using Knight's clock.
Lower right panel: The correlation between clinical and DNA methylation gestational age using Knight's clock calculated among n=380 newborns of mothers with optimal pregnancy dating based on having a regular menstrual cycle and a known first date of last menstrual period.
