# Supplementary material for: Associations of circulating folate, vitamin B12 and homocysteine concentrations in early pregnancy and cord blood with epigenetic gestational age: the Generation R Study
Source: Clin Epigenetics. 2021 Apr 29;13:95. doi: 10.1186/s13148-021-01065-x (PMC8082638; doi:10.1186/s13148-021-01065-x)
Supplement: Supplementary file 2 — Additional file 2: Table S1. Maternal and child characteristics based on imputed data (n = 1346). Table S2. Correlation matrix of circulating folate, vitamin B12 and homocysteine concentrations. Table S3. Non-response analysis. Table S4. Associations of maternal plasma homocysteine concentrations with raw gestational age acceleration estimated by Bohlin’s epigenetic clock (mediator models). Table S5. Associations of circulating folate, vitamin B12 and homocysteine concentrations with gestational age acceleration (reduced main models). Table S6. Associations of circulating folate, vitamin B12 and homocysteine concentrations with gestational age acceleration (crude models). Table S7. Associations of circulating folate, vitamin B12 and homocysteine concentrations with gestational age acceleration (basic models). Table S8. Associations of low versus normal early-pregnancy serum total B12 concentrations with gestational age acceleration. Table S9. Associations of cord serum active B12 concentrations with gestational age acceleration (sensitivity analysis). Table S10. Associations of cord serum vitamin B12 concentrations with gestational age acceleration estimated by Knight’s epigenetic clock (subgroup analysis, mediator models) [file 13148_2021_1065_MOESM2_ESM.docx]

**Associations of circulating folate, vitamin B12 and homocysteine concentrations in early pregnancy and cord blood with epigenetic gestational age: the Generation R Study**

***Additional file 2***

Giulietta S. Monasso^1,2^, Leanne K. Küpers^1,2,3^, Vincent W.V. Jaddoe^1,2^, Sandra G. Heil^4^, Janine F. Felix^1,2^

1. The Generation R Study Group, Erasmus MC, University Medical Center Rotterdam, Rotterdam, the Netherlands;
2. Department of Pediatrics, Erasmus MC, University Medical Center Rotterdam, Rotterdam, the Netherlands.
3. Division of Human Nutrition and Health, Wageningen University, Wageningen, The Netherlands
4. Department of Clinical Chemistry, Erasmus MC, University Medical Center Rotterdam, Rotterdam, the Netherlands.

**Content**

- **Tables: 10**

**Corresponding author:** Janine F. Felix, MD PhD, Generation R Study Group (Na-2918), Erasmus MC, University Medical Center Rotterdam, Rotterdam, the Netherlands. Phone: +31 10 7043405, fax: +31 10 70 44645, email: j.felix@erasmusmc.nl

**Table S1. Maternal and child characteristics based on imputed data (n=1346)^a^**

| **Maternal characteristics** |  |
| --- | --- |
| Age, year | 31.4 (4.2) |
| Highest completed education |  |
| No or Primary | 27 (2.0) |
| Secondary | 446 (32.9) |
| Higher | 881 (65.1) |
| Parity |  |
| Nulliparous | 821 (60.6) |
| Multiparous | 533 (39.4) |
| Pre-pregnancy BMI, kg/m^2^ | 22.4 (18.3, 33.3) |
| Smoking |  |
| Non-smoker or smoked until pregnancy was known | 1160 (85.7) |
| Smoked throughout pregnancy | 194 (14.3) |
| Gestational age at blood sampling, week | 12.8 (9.8, 16.9) |
| **Newborn Characteristics** |  |
| Gestational age at birth, weeks | 40.3 (36.7, 42.3) |
| DNA methylation gestational age (Bohlin), week | 39.4 (37.0, 40.8) |
| Raw gestational age acceleration (Bohlin), week | -0.9 (-2.7, 0.9) |
| DNA methylation gestational age (Knight), week | 36.5 (32.4, 39.1) |
| Raw gestational age acceleration (Knight), week | −3.7 (-7.4, -1.1) |
| Sex |  |
| Boy | 684 (50.8) |
| Girl | 662 (49.2) |
| Birth weight, gram | 3545 (510) |

a Values are mean (SD) or median (95% range) for continuous variables and numbers (%) for categorical variables. DNA methylation gestational age and gestational age acceleration estimated using Bohlin’s epigenetic clock was available in n=1335 newborns.

**Table S2. Correlation matrix of circulating folate, vitamin B12 and homocysteine concentrations ^a,b^**

|  | **Folate** | | **Total B12** | | **Active B12** | | **Homocysteine** | |
| --- | --- | --- | --- | --- | --- | --- | --- | --- |
|  | Maternal | Neonatal | Maternal | Neonatal | Maternal | Neonatal | Maternal | Neonatal |
| **Folate** |  |  |  |  |  |  |  |  |
| Maternal | 1.00 | 0.37 | 0.10 | 0.12 | 0.18 | 0.09 | -0.23 | -0.15 |
| Neonatal |  | 1.00 | 0.08 | 0.15 | 0.13 | 0.19 | -0.12 | -0.26 |
| **Total B12** |  |  |  |  |  |  |  |  |
| Maternal |  |  | 1.00 | 0.44 | 0.57 | 0.30 | -0.22 | -0.19 |
| Neonatal |  |  |  | 1.00 | 0.48 | 0.60 | -0.12 | -0.23 |
| **Active B12** |  |  |  |  |  |  |  |  |
| Maternal |  |  |  |  | 1.00 | 0.56 | -0.20 | -0.28 |
| Neonatal |  |  |  |  |  | 1.00 | -0.12 | -0.35 |
| **Homocysteine** |  |  |  |  |  |  |  |  |
| Maternal |  |  |  |  |  |  | 1.00 | 0.26 |
| Neonatal |  |  |  |  |  |  |  | 1.00 |

a Values represent Spearman correlation and are based on pairwise comparisons.

b Folate and homocysteine concentrations were measured in plasma and total and active B12 concentrations were measured in serum. We calculated standard deviation scores for all exposures to make them more comparable.

**Table S3. Non-response analysis (n=9901) ^a,b^**

|  | **Included**  **n=1346** | **Not included**  **n=8555** | ***P* value ^c^** |
| --- | --- | --- | --- |
| **Maternal characteristics** |  |  |  |
| Age, year | 31.4 (4.2) | 29.3 (5.5) | **< 0.001** |
| Highest completed education |  |  | **< 0.001** |
| No or Primary | 26 (2.0) | 942 (12.8) |  |
| Secondary | 439 (33.1) | 3541 (48.3) |  |
| Higher | 826 (65.0) | 2851 (38.9) |  |
| Parity |  |  | **< 0.001** |
| Nulliparous | 817 (60.7) | 4422 (54.1) |  |
| Multiparous | 528 (39.3) | 3755 (45.9) |  |
| Pre-pregnancy BMI, kg/m^2^ | 22.3 (18.4, 33.7) | 22.6 (17.8, 35.3) | **0.01** |
| Smoking |  |  | **< 0.001** |
| Non-smoker or smoked until pregnancy was known | 1059 (85.7) | 5782 (81.4) |  |
| Smoked throughout pregnancy | 176 (14.3) | 1325 (18.6) |  |
| Gestational age at blood sampling in pregnancy, week | 12.8 (9.9, 16.9) | 13.4 (9.5, 17.6) | **< 0.001** |
| Plasma folate concentrations, nmol/L | 19.8 (6.6, 39.6) | 14.7 (5.3, 37.1) | **< 0.001** |
| Serum total B12 concentrations, pmol/L | 178.0 (81.9, 428.5) | 167.0 (71.0, 405.1) | **< 0.001** |
| Serum active B12 concentrations, pmol/L | 44.0 (21.0, 94.0) | 41.0 (17.0, 97.8) | **< 0.001** |
| Plasma homocysteine concentrations, μmol/L | 7.0 (4.7, 11.5) | 6.9 (4.6, 12.1) | 0.70 |
| **Newborn Characteristics** |  |  |  |
| Gestational age at birth, week | 40.3 (36.7, 42.3) | 40.0 (34.6, 42.3) | **< 0.001** |
| Sex |  |  | 0.91 |
| Boy | 684 (50.8) | 4254 (50.6) |  |
| Girl | 662 (49.2) | 4146 (49.4) |  |
| Birth weight, gram | 3546 (510) | 3359 (594) | **< 0.001** |
| Plasma folate concentrations, nmol/L | 21.2 (11.0, 38.4) | 20.2 (10.2, 38.7) | **< 0.001** |
| Serum total B12 concentrations, pmol/L | 309.0 (128.0, 869.2) | 299.0 (117.0, 919.2) | 0.12 |
| Serum active B12 concentrations, pmol/L | 87.0 (39.0, 128.0) | 87.0 (35.0, 128.0) | 0.94 |
| Plasma homocysteine concentrations, μmol/L | 9.1 (5.5, 16.4) | 9.1 (5.1, 16.7) | 0.41 |

a The non-response analysis compared the 1346 newborns who were included in the analyses to those who participated in Generation R at birth but were not included (n=8555), because either no DNA methylation was measured (n=8505), or information on all exposures was missing (n=35), or their sibling was included in the analyses (n=15).

b Values are based on non-imputed data and are mean (SD) or median (95% range) for continuous variables and numbers (%) for categorical variables. We calculated standard deviation scores for all exposures to make them more comparable.

c P values for differences in subject characteristics between groups were calculated performing independent sample t-tests (normally distributed continuous variables), Mann Whitney tests (not normally distributed continuous variables) and chi-square tests (categorical variables).

**Table S4. Associations of maternal plasma homocysteine concentrations with raw gestational age acceleration estimated by Bohlin’s clock (mediator models) ^a,b,c^**

|  | **Birth weight model** | | **Folate model** | | | **Total B12 model** | | | **Active B12 model** | | |  |
| --- | --- | --- | --- | --- | --- | --- | --- | --- | --- | --- | --- | --- |
|  | **Difference (95% CI) in weeks** | ***P* value** | | **Difference (95% CI) in weeks** | ***P* value** | | **Difference (95% CI) in weeks** | ***P* value** | | **Difference (95% CI) in weeks** | ***P* value** | |
| Homocysteine, SDS | 0.07 (-0.02, 0.15) | 0.11 | | 0.05 (-0.06, 0.16) | 0.90 | | 0.08 (-0.01. 0.17) | 0.08 | | 0.10 (-0.02. 0.20) | 0.06 | |

Abbreviations: CI, confidence interval; SDS, standard deviation score.

a The full study population included n=1335 mother-newborn pairs after exclusion of 11 newborns with missing information on Bohlin’s DNA methylation gestational age. Values represent regression coefficients (95% confidence interval) and reflect the difference in raw and residual gestational age acceleration at birth per increase of 1 standard deviation score in exposure variable.

b Results are based on the mediator models, which were adjusted for maternal age, education, pre-pregnancy BMI, parity and smoking, child sex, batch effects (by including plate number), cell types and additionally for birth weight, folate, total B12 or active B12, respectively. The mediator models were only run in case of significant associations in the main models.

c Raw gestational age acceleration (in weeks) was obtained by subtracting the clinical estimate of gestational age from DNA methylation gestational age.

**Table S5. Associations of circulating folate, vitamin B12 and homocysteine concentrations with gestational age acceleration (reduced main models) ^a,b^**

|  | **Bohlin** | | | | | **Knight** | | | | | |
| --- | --- | --- | --- | --- | --- | --- | --- | --- | --- | --- | --- |
|  | **Raw acceleration ^c^** | | **Residual acceleration ^d^** | | | **Raw acceleration ^c^** | | | **Residual acceleration ^d^** | | |
|  | **Difference (95% CI) in weeks** | ***P* value** | | **Difference (95% CI)** | ***P* value** | | **Difference (95% CI) in weeks** | ***P* value** | | **Difference (95% CI)** | ***P* value** |
| **Early pregnancy** |  |  | |  |  | |  |  | |  |  |
| Folate, SDS | 0.03 (-0.03, 0.08) | 0.37 | | -0.00 (-0.04, 0.03) | 0.87 | | -0.01 (-0.10, 0.08) | 0.88 | | -0.03 (-0.11, 0.05) | 0.50 |
| Total B12, SDS | -0.02 (-0.07, 0.04) | 0.60 | | 0.01 (-0.03, 0.05) | 0.55 | | -0.03 (-0.12, 0.06) | 0.48 | | -0.01 (-0.09, 0.07) | 0.80 |
| Active B12, SDS | -0.04 (-0.10, 0.03) | 0.29 | | -0.07 (-0.16, 0.03) | 0.16 | | -0.09 (-0.19, 0.02) | 0.10 | | -0.07 (-0.16, 0.03) | 0.16 |
| Homocysteine, SDS | **0.07 (0.02, 0.12)** | **0.01*** | | 0.01 (-0.02, 0.05) | 0.54 | | 0.07 (-0.02, 0.16) | 0.11 | | 0.03 (-0.05, 0.10) | 0.50 |
| **Cord blood** |  |  | |  |  | |  |  | |  |  |
| Folate, SDS | 0.02 (-0.03, 0.08) | 0.86 | | 0.01 (-0.03, 0.04) | 0.64 | | 0.03 (-0.06, 0.12) | 0.49 | | 0.02 (-0.06, 0.10) | 0.67 |
| Total B12, SDS | -0.04 (-0.09, 0.01) | 0.14 | | 0.03 (-0.00, 0.06) | 0.07 | | -0.06 (-0.14, 0.03) | 0.20 | | 0.01 (-0.07, 0.08) | 0.89 |
| Active B12, SDS | 0.02 (-0.03, 0.08) | 0.40 | | 0.00 (-0.08, 0.08) | 1.00 | | 0.00 (-0.09, 0.09) | 0.99 | | 0.00 (-0.08, 0.08) | 1.00 |
| Homocysteine, SDS | -0.01 (-0.06, 0.05) | 0.79 | | -0.02 (-0.06, 0.01) | 0.23 | | 0.03 (-0.06, 0.11) | 0.53 | | 0.02 (-0.06, 0.09) | 0.70 |

Abbreviations: CI, confidence interval; SDS, standard deviation score.

a The full study population included n=1346 mother-newborn pairs. Values represent regression coefficients (95% confidence interval) and reflect the difference in raw and residual gestational age acceleration at birth per increase of 1 standard deviation score in exposure variable. Results are based on the main models but not adjusted for cell type proportions. The models were adjusted for maternal age, education, pre-pregnancy BMI, parity and smoking, child sex, batch effects (by including plate number) and additionally for gestational age at blood sampling in early pregnancy models.

b Folate and homocysteine concentrations were measured in plasma and total and active B12 concentrations were measured in serum.

c Raw gestational age acceleration (in weeks) was obtained by subtracting the clinical estimate of gestational age from DNA methylation gestational age.

d Residual gestational age acceleration (no unit) was calculated from the residuals from a regression model of DNA methylation gestational age on clinical gestational age.

**Table S6. Associations of circulating folate, vitamin B12 and homocysteine concentrations with gestational age acceleration (crude models) ^a,b^**

|  | **Bohlin** | | | | | **Knight** | | | | | |
| --- | --- | --- | --- | --- | --- | --- | --- | --- | --- | --- | --- |
|  | **Raw acceleration ^c^** | | **Residual acceleration ^d^** | | | **Raw acceleration ^c^** | | | **Residual acceleration ^d^** | | |
|  | **Difference (95% CI) in weeks** | ***P* value** | | **Difference (95% CI)** | ***P* value** | | **Difference (95% CI) in weeks** | ***P* value** | | **Difference (95% CI)** | ***P* value** |
| **Early pregnancy** |  |  | |  |  | |  |  | |  |  |
| Folate, SDS | 0.01 (-0.05, 0.06) | 0.79 | | -0.01 (-0.04, 0.03) | 0.74 | | -0.01 (-0.10, 0.08) | 0.82 | | -0.02 (-0.10, 0.06) | 0.64 |
| Total B12, SDS | -0.02 (-0.08, 0.03) | 0.43 | | 0.01 (-0.03, 0.04) | 0.66 | | -0.03 (-0.12, 0.05) | 0.45 | | -0.01 (-0.09, 0.07) | 0.83 |
| Active B12, SDS | -0.03 (-0.10, 0.03) | 0.29 | | -0.06 (-0.16, 0.03) | 0.19 | | -0.08 (-0.18, 0.02) | 0.12 | | -0.06 (-0.16, 0.03) | 0.19 |
| Homocysteine, SDS | **0.08 (0.03, 0.14)** | **0.003*** | | 0.02 (-0.02, 0.05) | 0.37 | | 0.08 (-0.01, 0.16) | 0.08 | | 0.02 (-0.05, 0.20) | 0.55 |
| **Cord blood** |  |  | |  |  | |  |  | |  |  |
| Folate, SDS | 0.02 (-0.04, 0.07) | 0.55 | | 0.01 (-0.03, 0.04) | 0.71 | | 0.03 (-0.05, 0.12) | 0.47 | | 0.02 (-0.06, 0.10) | 0.57 |
| Total B12, SDS | -0.04 (-0.09, 0.01) | 0.13 | | 0.03 (-0.00, 0.06) | 0.08 | | -0.05 (-0.13, 0.03) | 0.23 | | 0.01 (-0.07, 0.08) | 0.81 |
| Active B12, SDS | 0.02 (-0.03, 0.08) | 0.46 | | 0.01 (-0.07, 0.08) | 0.90 | | 0.00 (-0.08, 0.09) | 0.96 | | 0.01 (-0.07, 0.08) | 0.90 |
| Homocysteine, SDS | -0.00 (-0.06, 0.05) | 0.91 | | -0.02 (-0.05, 0.02) | 0.27 | | 0.03 (-0.06, 0.12) | 0.48 | | 0.02 (-0.06, 0.10) | 0.67 |

Abbreviations: CI, confidence interval; SDS, standard deviation score.

a The full study population included n=1346 mother-newborn pairs. Values represent regression coefficients (95% confidence interval) and reflect the difference in raw and residual gestational age acceleration at birth per increase of 1 standard deviation score in exposure variable. Results are based on the crude models and were adjusted for child sex, batch effects (by including plate number) and additionally for gestational age at blood sampling in early pregnancy models.

b Folate and homocysteine concentrations were measured in plasma and total and active B12 concentrations were measured in serum.

c Raw gestational age acceleration (in weeks) was obtained by subtracting the clinical estimate of gestational age from DNA methylation gestational age.

d Residual gestational age acceleration (no unit) was calculated from the residuals from a regression model of DNA methylation gestational age on clinical gestational age.

**Table S7. Associations of circulating folate, vitamin B12 and homocysteine concentrations with gestational age acceleration (basic models) ^a,b^**

|  | **Bohlin** | | | | | **Knight** | | | | | |
| --- | --- | --- | --- | --- | --- | --- | --- | --- | --- | --- | --- |
|  | **Raw acceleration ^c^** | | **Residual acceleration ^d^** | | | **Raw acceleration ^c^** | | | **Residual acceleration ^d^** | | |
|  | **Difference (95% CI) in weeks** | ***P* value** | | **Difference (95% CI)** | ***P* value** | | **Difference (95% CI) in weeks** | ***P* value** | | **Difference (95% CI)** | ***P* value** |
| **Early pregnancy** |  |  | |  |  | |  |  | |  |  |
| Folate, SDS | 0.01 (-0.05, 0.06) | 0.83 | | 0.00 (-0.03, 0.03) | 0.98 | | -0.01 (-0.09, 0.08) | 0.91 | | -0.01 (-0.08, 0.08) | 0.83 |
| Total B12, SDS | -0.02 (-0.07, 0.04) | 0.53 | | 0.00 (-0.03, 0.04) | 0.88 | | -0.04 (-0.13, 0.04) | 0.33 | | -0.02 (-0.10, 0.05) | 0.51 |
| Active B12, SDS | -0.03 (-0.10, 0.03) | 0.31 | | -0.02 (-0.11, 0.07) | 0.67 | | -0.05 (-0.14, 0.05) | 0.37 | | -0.02 (-0.11, 0.07) | 0.67 |
| Homocysteine, SDS | **0.07 (0.01, 0.12)** | **0.01*** | | 0.02 (-0.01, 0.05) | 0.29 | | 0.08 (0.00, 0.16) | 0.05 | | 0.03 (-0.05, 0.10) | 0.56 |
| **Cord blood** |  |  | |  |  | |  |  | |  |  |
| Folate, SDS | 0.02 (-0.04, 0.07) | 0.59 | | 0.01 (-0.03, 0.04) | 0.73 | | 0.04 (-0.04, 0.13) | 0.31 | | 0.04 (-0.04, 0.11) | 0.35 |
| Total B12, SDS | -0.03 (-0.09, 0.02) | 0.21 | | 0.02 (-0.01, 0.05) | 0.23 | | -0.03 (-0.11, 0.05) | 0.46 | | 0.02 (-0.06, 0.09) | 0.66 |
| Active B12, SDS | 0.02 (-0.03, 0.08) | 0.41 | | 0.01 (-0.06, 0.09) | 0.73 | | 0.02 (-0.07, 0.10) | 0.67 | | 0.01 (-0.06, 0.09) | 0.73 |
| Homocysteine, SDS | 0.00 (-0.05, 0.06) | 0.91 | | -0.01 (-0.04, 0.02) | 0.48 | | 0.04 (-0.05, 0.12) | 0.41 | | 0.02 (-0.05, 0.10) | 0.57 |

Abbreviations: CI, confidence interval; SDS, standard deviation score.

a The full study population included n=1346 mother-newborn pairs. Values represent regression coefficients (95% confidence interval) and reflect the difference in raw and residual gestational age acceleration at birth per increase of 1 standard deviation score in exposure variable. Results are based on the basic models and were adjusted for child sex, batch effects (by including plate number), cell types, and additionally for gestational age at blood sampling in early pregnancy models.

b Folate and homocysteine concentrations were measured in plasma and total and active B12 concentrations were measured in serum.

c Raw gestational age acceleration (in weeks) was obtained by subtracting the clinical estimate of gestational age from DNA methylation gestational age.

d Residual gestational age acceleration (no unit) was calculated from the residuals from a regression model of DNA methylation gestational age on clinical gestational age.

**Table S8. Associations of low versus normal early pregnancy serum total B12 concentrations with gestational age acceleration ^a,b^**

|  | **Bohlin** | | | | | **Knight** | | | | | |
| --- | --- | --- | --- | --- | --- | --- | --- | --- | --- | --- | --- |
|  | **Raw acceleration ^c^** | | **Residual acceleration ^d^** | | | **Raw acceleration ^c^** | | | **Residual acceleration ^d^** | | |
|  | **Difference (95% CI) in weeks** | ***P* value** | | **Difference (95% CI)** | ***P* value** | | **Difference (95% CI) in weeks** | ***P* value** | | **Difference (95% CI)** | ***P* value** |
| Total B12 ≥145 pmol/L | *Reference* |  | | *Reference* |  | | *Reference* |  | | *Reference* |  |
| Total B12 <145 pmol/L | 0.03 (-0.09, 0.16) | 0.59 | | 0.01 (-0.06, 0.08) | 0.83 | | 0.05 (-0.14, 0.23) | 0.64 | | 0.02 (-0.15, 0.18) | 0.83 |

Abbreviations: CI, confidence interval; SDS, standard deviation score.

a In the full study population n=1036 mothers had information on serum total B12 concentrations available. Values represent regression coefficients (95% confidence interval) and reflect the difference in raw and residual gestational age acceleration at birth between children of mothers with total B12 concentrations in early pregnancy below the 95% reference interval for healthy adults, as compared to children of mothers with total B12 concentrations within this range. Results are based on the main models which were adjusted for maternal age, education, pre-pregnancy BMI, parity and smoking, child sex, batch effects (by including plate number), cell types and gestational age at blood sampling.

b For the other exposures, the distribution of mothers or newborns after dichotomization was deemed too uneven for meaningful analyses, and therefore these analyses were not conducted.

c Raw gestational age acceleration (in weeks) was obtained by subtracting the clinical estimate of gestational age from DNA methylation gestational age.

d Residual gestational age acceleration (no unit) was calculated from the residuals from a regression model of DNA methylation gestational age on clinical gestational age.

**Table S9. Associations of cord serum active B12 concentrations with gestational age acceleration (sensitivity analysis) ^a,b^**

|  | **Bohlin** | | | | | **Knight** | | | | | |
| --- | --- | --- | --- | --- | --- | --- | --- | --- | --- | --- | --- |
|  | **Raw acceleration ^c^** | | **Residual acceleration ^d^** | | | **Raw acceleration ^c^** | | | **Residual acceleration ^d^** | | |
|  | **Difference (95% CI) in weeks** | ***P* value** | | **Difference (95% CI)** | ***P* value** | | **Difference (95% CI) in weeks** | ***P* value** | | **Difference (95% CI)** | ***P* value** |
| Active B12, SDS | 0.05 (-0.02, 0.12) | 0.19 | | 0.04 (-0.01, 0.08) | 0.10 | | 0.01 (-0.11, 0.12) | 0.92 | | -0.01 (-0.10, 0.09) | 0.92 |

Abbreviations: CI, confidence interval; SDS, standard deviation score.

a In the full study population n=189 newborns had cord serum active B12 concentrations recorded as the upper limit of the immuno-assay for active B12. After excluding these newborns, the sensitivity analysis included n=937 newborns with information on cord serum active B12 concentrations. New distribution of active B12 concentrations, median (95% range), pmol/L: 80.0 (37.0, 124.0). For the other exposures we did not perform similar sensitivity analyses because only few subjects had concentrations corresponding to the limit of the analytic range of the immuno-assay: maternal folate: n=9, neonatal folate: n=8; maternal total B12: n=1, neonatal total B12: n=2; maternal active B12: n=4; maternal and neonatal homocysteine: n=0.

b Values represent regression coefficients (95% confidence interval) and reflect the difference in raw and residual gestational age acceleration at birth per increase of 1 standard deviation score in active B12 concentrations. Results are based on the main models, which were adjusted for maternal age, education, pre-pregnancy BMI, parity and smoking, child sex, batch effects (by including plate number), cell types.

c Raw gestational age acceleration (in weeks) was obtained by subtracting the clinical estimate of gestational age from DNA methylation gestational age.

d Residual gestational age acceleration (no unit) was calculated from the residuals from a regression model of DNA methylation gestational age on clinical gestational age.

**Table S10. Associations of cord serum vitamin B12 concentrations with raw gestational age acceleration estimated by Knight’s clock (subgroup analysis, mediator models) ^a,b,c^**

|  | **Birth weight model** | | **Folate model** | | **Homocysteine model** | |
| --- | --- | --- | --- | --- | --- | --- |
|  | **Difference (95% CI) in weeks** | ***P* value** | **Difference (95% CI) in weeks** | ***P* value** | **Difference (95% CI) in weeks** | ***P* value** |
| Total B12, SDS | -0.18 (-0.32, -0.04) | **0.01*** | -0.19 (-0.34, -0.05) | **0.009*** | -0.18 (-0.32, -0.03) | **0.02*** |
| Active B12, SDS | -0.15 (-0.29, -0.01) | **0.04*** | -0.17 (-0.32, -0.02) | **0.03*** | -0.16 (-0.32, 0.004) | 0.06 |

Abbreviations: CI, confidence interval; SDS, standard deviation score.

a This subgroup analysis included 380 newborns of mothers with optimal pregnancy dating based on a regular menstrual cycle and gestational age determined by last menstrual period. Values represent regression coefficients (95% confidence interval) and reflect the difference in raw and residual gestational age acceleration at birth per increase of 1 standard deviation score in vitamin B12 concentrations.

b Results are based on the mediator models, which were adjusted for maternal age, education, pre-pregnancy BMI, parity and smoking, child sex, batch effects (by including plate number), cell types and additionally for birth weight, folate or homocysteine, respectively. The mediator models were only run in case of significant associations in the main models.

c Raw gestational age acceleration (in weeks) was obtained by subtracting the clinical estimate of gestational age from DNA methylation gestational age.
